# Cell Death in Orthodontic Tooth Movement: Recent Advances and Emerging Insights

**DOI:** 10.3390/ijms27021130

**Published:** 2026-01-22

**Authors:** Fumitoshi Ohori, Hideki Kitaura, Aseel Marahleh, Jinghan Ma, Kohei Narita, Angyi Lin, Ziqiu Fan, Kou Murakami, Hiroyasu Kanetaka

**Affiliations:** 1 Division of Orthodontics and Dentofacial Orthopedics, Graduate School of Dentistry, Tohoku University, Sendai 980-8575, Miyagi, Japan; aseel.mahmoud.suleiman.marahleh.e6@tohoku.ac.jp (A.M.); ma.jinghan.c1@tohoku.ac.jp (J.M.); kohei.narita.a2@tohoku.ac.jp (K.N.); lin.angyi.r5@dc.tohoku.ac.jp (A.L.); fan.ziqiu.q1@dc.tohoku.ac.jp (Z.F.); kou.murakami.b2@tohoku.ac.jp (K.M.); hiroyasu.kanetaka.e6@tohoku.ac.jp (H.K.); 2 Creative Interdisciplinary Research Division, Frontier Research institute for Interdisciplinary Sciences, Tohoku University, Sendai 980-0845, Miyagi, Japan; 3 Division of Advanced Dental Science and Technology, Graduate School of Biomedical Engineering, Tohoku University, Sendai 980-8579, Miyagi, Japan

**Keywords:** cell death, orthodontic tooth movement, bone remodeling, periodontal ligament, cementoblast, cementocyte, osteocyte, osteoblast, osteoclast, root resorption

## Abstract

Orthodontic tooth movement (OTM), a complex biological process driven by orchestrated bone remodeling, involves osteoclastic bone resorption and osteoblastic bone formation in response to mechanical force. Traditionally, OTM-related cell death has been discussed in terms of apoptosis and necrosis. However, recent advances in cell death research have revealed various forms of regulated cell death (RCD) beyond these conventional categories. This review summarizes the current understanding of the diverse RCD pathways and their roles in various cell populations during OTM. It delineates the involvement of distinct RCD mechanisms, including apoptosis, autophagy, pyroptosis, ferroptosis, and necroptosis. On the compression side, these RCD pathways in periodontal ligament (PDL) cells, cementoblasts, cementocytes, and bone-related cells actively drive inflammatory responses, promote bone resorption, and contribute to root resorption. Conversely, on the tension side, specific RCD pathways, notably autophagy in the PDL and osteocytes, play crucial roles in promoting osteogenesis and tissue repair. Collectively, cell death is not merely a passive elimination of cells but actively functions as a critical switch for alveolar bone remodeling during OTM. Understanding these multifaceted RCD mechanisms provides novel insights into the biological regulation of tooth movement and identifies potential therapeutic targets for enhancing tooth movement efficiency and mitigating adverse effects.

## 1. Introduction

**Figure 1:**
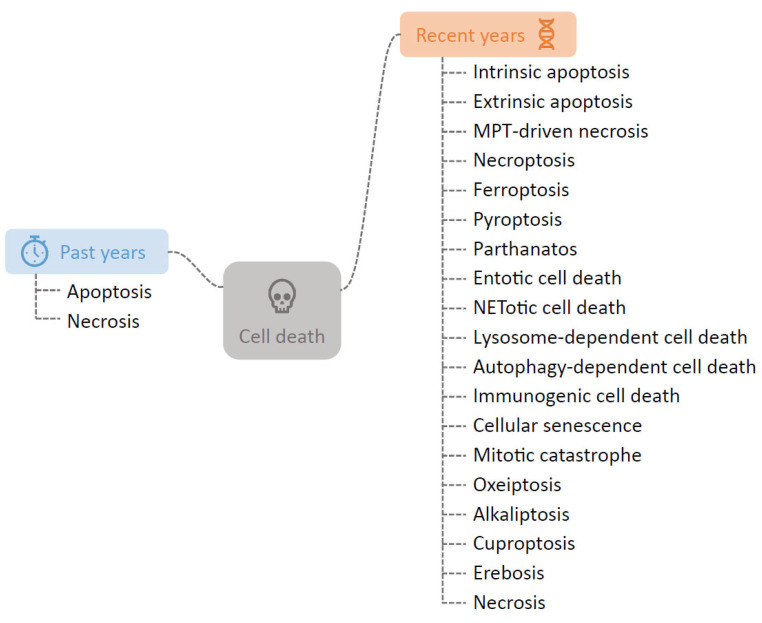
Overview of expanding landscape of cell death. The schema illustrates the transition from the historical classification (**left**) to the contemporary multifaceted framework (**right**). Each branch represents a distinct form of cell death, including both established categories defined by the NCCD and emerging types identified in recent literature. These various types are classified based on their unique molecular, biochemical, or morphological characteristics.

Cell death is an essential biological process in multicellular organisms. Although the word “death” generally carries a negative connotation, “cell death” is crucial for maintaining homeostasis and ensuring precise biological reactions. Historically, cell death has been broadly categorized into two types: apoptosis, a form of programmed cell death (PCD), and necrosis, traditionally considered an unregulated and accidental cell death (ACD) [1]. However, advances in cell death research have revealed a wide variety of regulated cell death (RCD) processes beyond these traditional categories. As cell death mechanisms have become more complex and varied, the Nomenclature Committee on Cell Death (NCCD) was established to create standard definitions and classifications of cell death [2]. In 2018, the NCCD proposed a new classification system for cell death, which was based on molecular mechanisms rather than morphological features. This updated framework includes various RCDs, including intrinsic apoptosis, extrinsic apoptosis, mitochondrial permeability transition (MPT)-driven necrosis, necroptosis, ferroptosis, pyroptosis, parthanatos, entotic cell death, NETotic cell death, lysosome-dependent cell death, autophagy-dependent cell death, immunogenic cell death, cellular senescence, and mitotic catastrophe [3]. More recently, other novel types of RCD have been discovered, including oxeiptosis, alkaliptosis, cuproptosis, and erebosis (Figure 1) [4,5,6,7]. Some of these forms have been identified in periodontal ligament cells and are closely related to periodontal tissue maintenance and remodeling [8].

Orthodontic tooth movement (OTM) is mediated by bone remodeling, which involves osteoclastic bone resorption and osteoblastic bone formation [9]. Osteoclasts are recruited to the compression side, where the periodontal ligament is mechanically compressed, whereas osteoblasts are activated on the tension side, where the periodontal ligament is stretched. These coordinated activities gradually shift the teeth toward new positions. A proper understanding of OTM requires a detailed knowledge of osteoclast and osteoblast differentiation. When orthodontic forces are applied, periodontal tissues detect mechanical stress and initiate a cascade of biological responses [10]. During OTM, various cytokines are released within the periodontal tissues and are detectable in the gingival crevicular fluid [11]. These cytokines regulate the differentiation and activity of osteoclasts and osteoblasts, and recent studies have further clarified their roles [12,13]. However, the involvement of cell death in the regulation of bone remodeling during OTM remains controversial. A central unresolved question is whether cell death acts as a primary initiator of bone remodeling or occurs as a secondary consequence of mechanical stress and inflammatory processes. This review aims to provide a critical evaluation and comparative analysis of current literature to address these conflicting perspectives. By synthesizing diverse experimental data, we seek to clarify the functional significance of various cell death pathways and their specific contributions to the complex process of orthodontic tissue changes.

## 2. Types of Cell Death During OTM

Various forms of cell death have been reported during OTM, each influencing the biological responses to mechanical forces. Although classifying the exact type of cell death occurring during OTM according to the current NCCD classification remains challenging, several types of cell death, including apoptosis and necrosis, autophagy, pyroptosis, ferroptosis, and necroptosis, have been reported. Hyalinized tissue formation on the compression side is a type of cell death associated with mechanical stress [14]. However, this review focuses on the recent insights regarding the specific cell death mechanisms in individual cell populations involved in OTM, apart from the phenomenon of hyalinized tissues.

### 2.1. Apoptosis and Necrosis

Apoptosis is a form of RCD involving the activation of caspase family proteases, including caspase-3, -6, -7, -8, and -9 [15]. It is morphologically characterized by cell shrinkage, chromatin condensation, and formation of small membrane-bound apoptotic bodies, which are subsequently phagocytosed by neighboring parenchymal cells, neoplastic cells, or macrophages [16]. Apoptosis is generally classified into two major pathways: intrinsic apoptosis initiated by permeabilization of the outer mitochondrial membrane and mediated by pro-apoptotic members of the B-cell lymphoma 2 (Bcl-2) family; and extrinsic apoptosis triggered by the activation of cell surface death receptors, such as tumor necrosis factor (TNF) and FS-7-associated surface antigen (FAS) receptors, upon ligand binding. In contrast, necrosis is a form of accidental cell death (ACD) occurring in response to factors such as physical injury, trauma, or infection [3]. Necrosis is characterized by cell swelling, plasma membrane rupture, and release of intracellular contents, which trigger inflammation and tissue damage [17].

The initial observation of apoptosis in cells during OTM involves osteoclasts. In growing rats, the physiological distal movement of maxillary first molars is associated with numerous osteoclasts on the distal alveolar bone surface. When maxillary first molars are moved mesially in an experimental rat model of tooth movement, the distal alveolar bone surface quickly shifts from bone resorption to bone formation. This process is accompanied by the disappearance of existing osteoclasts, which show characteristic morphological signs of apoptosis [18]. Furthermore, osteoclast clearance on the compression side during OTM is also mediated by apoptosis. Thus, the experimental model of tooth movement in rats demonstrates a highly significant increase in apoptotic osteoclast nuclei in the treated groups compared with those in the control groups, suggesting that apoptosis is a key mechanism for clearing the osteoclasts recruited for tooth movement [19].

#### 2.1.1. Apoptosis in Periodontal Ligament (PDL) Cells

An investigation using the terminal deoxynucleotidyl transferase-mediated dUTP nick end labeling (TUNEL) assay to detect apoptosis during OTM in rats showed a significant increase in TUNEL-positive cells after 3 days of orthodontic force application. Although apoptotic cells were not specifically identified, the results confirmed that apoptosis was a critical response to OTM [20]. In a study investigating the cellular responses of periodontal ligaments (PDL) during OTM in rats, cell proliferation, detected using proliferating cell nuclear antigen (PCNA), was found to peak on the tension side after 3 days of OTM. In contrast, apoptosis, as detected by TUNEL staining, increased continuously on both the compression and tension sides throughout the OTM period. These findings indicate that cell proliferation and death are precisely regulated during PDL regeneration and reconstruction during OTM [21]. In the OTM rat model, TUNEL-positive PDL cells began to appear on the compression side 12 h after force application, peaking at 24 h. The TUNEL-positive PDL cells then disappeared at 48 h, immediately followed the initiation of alveolar bone resorption at 72 h. These results demonstrate that PDL cell apoptosis is a critical early-phase event preceding alveolar bone resorption [22]. Zhang et al. investigated the relationship between the mechanical stretching force and apoptosis in human PDL cells in vitro. They demonstrated that stretching force induces early apoptosis in human PDL cells through caspase-9 activation [23]. Furthermore, quantitative real-time array analysis revealed several force-sensing genes involved in the apoptotic pathways in human PDL cells subjected to stretching force [24].

In addition to identifying apoptosis using TUNEL staining, the molecular basis of apoptosis on the compression side has also been gradually elucidated. A study focusing on the compression side in OTM model rats confirmed an increase in TUNEL and caspase-8 positive cells in vivo. A crucial mechanism was revealed in vitro, showing that compression force induces both G1 arrest and apoptosis in human PDL cells, primarily inducing the production of intracellular reactive oxygen species (ROS), which subsequently activate the apoptotic pathway [25]. A previous study showed that compressive force rapidly triggered apoptosis in both PDL and vascular endothelial cells in occlusal hypofunction, demonstrating that the inclination of hypofunctional teeth increased more than that of normal teeth during OTM [26]. In a mouse orthodontic relapse model, in which closed-coil springs were removed after moving the maxillary first molar mesially, PDL apoptosis increased on the compression side (mesial side) in the OTM group and on the relapse-compression side (distal side) in the relapse group [27]. Micro-osteoperforations (MOPs) are known to accelerate tooth movement. A relevant mechanistic report on a rat OTM model showed that MOPs stimulated PDL cell cycles, increasing PCNA- and TUNEL-positive PDL cells, thereby promoting tooth movement without exacerbating the progression of root resorption [28,29]. The same research group also investigated the mechanism of tooth movement acceleration caused by a vibration stimulus. The vibration stimulus enhanced PDL cell proliferation and apoptosis on the compression side and was effective at promoting tooth movement [30]. In contrast, the cellular response of the PDL to a simple compressive force may be decelerated. For instance, an in vitro study on human PDL fibroblasts showed that mechanical compression significantly reduced cell proliferation and total cell number, with a higher rate of apoptosis [31]. A recent report suggests that integrin-linked kinase regulates PDL cell proliferation and apoptosis, making them potential targets for accelerating tooth movement [32]. Kaya et al. further reported the effects of orthodontic light (10 cN) and heavy (60 cN) forces on PDL cell apoptosis. They showed that heavy forces promoted apoptosis of PDL cells. Conversely, tooth movement rates were the same for both light and heavy forces [33]. Research on the anti-apoptotic molecular mechanisms of superoxide dismutase 2 (SOD2) and baculoviral IAP repeat-containing protein 3 (BIRC3) indicates that, while periodontal infection upregulates these molecules as a protective mechanism, concomitant application of orthodontic force significantly inhibits the increase in SOD2 and BIRC3 both in vivo and in vitro. These results suggest that mechanical loading compromises an essential periodontal anti-apoptotic defense system [34]. The mechanosensitive ion channel, Piezo1, has been implicated in force-induced cell death, including apoptosis and ferroptosis [35]. A study using a rat OTM model in vivo and compressive loading in vitro demonstrated that Piezo1 mediates apoptosis in PDL fibroblasts. Specifically, inhibiting Piezo1 expression using GsMTx4 significantly alleviated force-induced apoptosis, confirming the regulatory role of Piezo1 through activation of the p38/ERK1/2 signaling pathway during OTM [36]. A recent study further elucidated the role of apoptosis in bone regeneration during OTM. Liu et al. identified leptin receptor-positive cells (Lepr+) as distinct osteoprogenitors activated by force-induced PDL cell apoptosis. Specifically, apoptotic vesicles from PDL stem cells were found to activate the differentiation of Lepr+ cells into osteoblasts, whereas inhibiting apoptosis downregulated this osteogenesis. These findings revealed that PDL apoptosis serves as a signal that triggers alveolar bone formation [37]. Collectively, studies on PDL cells indicate that apoptosis is a critical early event on the compression side that precedes the initiation of alveolar bone resorption. This timing suggests that PDL cell elimination is a carefully regulated process that initiates the tissue turnover necessary for alveolar bone remodeling, thereby indicating a potential role in mediating and regulating OTM.

#### 2.1.2. Apoptosis in Cementoblasts and Cementocytes

Apoptosis extends to the root surface and compressive forces alone or in combination with interleukin 1 beta (IL-1β) stimulation are known to induce apoptosis-related gene expression and caspase activity in human primary cementoblasts [38]. Further mechanistic investigation has confirmed that inhibition of platelet-derived growth factor receptor-β (PDGFRβ) signaling increases the expression of pro-apoptotic gene under compressive force in human primary cementoblasts. These reports suggest a protective role for the PDGF signaling pathway in regulating cementoblast viability and potentially mitigating root resorption during OTM [39]. As cementoblast apoptosis may contribute to the impaired repair of root resorption induced by orthodontic force, the role of AXUD1, a novel pro-apoptotic gene, was investigated. The results indicated that compressive force upregulated AXUD1 expression in human primary cementoblasts in a time- and force-dependent manner. Notably, siRNA-mediated knockdown of AXUD1 reduced apoptosis and decreased JNK phosphorylation, thereby establishing the AXUD1-JNK pathway as a key molecular mechanism underlying cementoblast apoptosis [40]. In vitro analysis of the compressive force applied to cementoblast-like cells revealed that compressive force induces caspases 3 and 8, which subsequently promote receptor activator of nuclear factor κB ligand (RANKL) expression in these cells [41]. The clinical relevance of this mechanism was validated in vivo using a rat OTM model, in which a heavy orthodontic force (50 g) was found to induce caspase 3- and 8-positive cells on the cementum surface. These findings strongly confirm that cementoblast apoptosis is a critical event in the pathology of orthodontic root resorption [42]. The role of hypoxic conditions (often secondary to compression in OTM) has also been explored. Hypoxia-induced apoptosis in cementoblats is mediated by hypoxia inducible factor 1α (HIF-1α), which acts as a bridge factor for ERK1/2 and caspases. Adipokines such as resistin, visfatin, and ghrelin inhibit hypoxia-induced apoptosis via ERK1/2 dependent upregulation of HIF-1α, suggesting their potential as molecular targets for orthodontic treatment in patients with obesity [43].

Although fewer studies have been conducted on cementocyte apoptosis than on cementoblasts, the available evidence clearly confirms its occurrence during OTM. For instance, Matsuzawa et al. demonstrated that cementocytes adjacent to the hyalinized periodontal tissue underwent apoptotic cell death during OTM in rats. Notably, cleaved caspase-3- and ssDNA-positive cementocytes appeared at an early stage after force application, preceding the appearance of odontoclasts and subsequent root resorption [44]. In a subsequent study, Santos et al. investigated the ultrastructural and proteomic alterations of cementocytes and cellular cementum during OTM in rats. A small number of cementocytes demonstrated apoptotic features associated with localized areas of root resorption. Proteomic analysis further revealed the downregulation of several extracellular matrix proteins, indicating that mechanical loading induces both apoptotic and matrix remodeling responses in cementocytes [45]. Collectively, these results highlight the importance of apoptosis in cementoblasts and cementocytes as a critical pathological mechanism that precedes and promotes the initiation of orthodontic force-induced root resorption.

#### 2.1.3. Apoptosis and Necrosis in Bone-Related Cells

Finally, this section details the roles of apoptosis and necrosis in bone-related cells, including osteocytes, osteoblasts, and osteoclasts. Hamaya et al. were the first to report osteocyte death in the alveolar bone in a rat OTM model. According to this report, osteocyte apoptosis is initiated rapidly within 6 h after force application, followed by a peak in osteocyte necrosis on day 2 and osteocyte disappearance (empty lacunae) on day 4 of OTM [46]. A study using different force magnitudes (light: 10–15 g or heavy: 20–25 g) in a rat OTM model demonstrated that osteocyte apoptosis on the compression side increased rapidly by day 1 and reached a plateau in both groups, indicating that osteocyte apoptosis was independent of the applied orthodontic force [47]. Contrary to in vivo observations, Tan et al. demonstrated that pulsating fluid flow, simulating mechanical loading, inhibited TNF-α-induced apoptosis in osteocytes in vitro. This finding suggests that the mechanosensing ability of osteocytes provides a protective signal that helps regulate cell death and subsequent bone remodeling under orthodontic force [48]. A study using a mouse OTM model found a sequential relationship between connective tissue growth factor (CTGF) and apoptosis in osteocytes, suggesting that CTGF signaling may play a key role in triggering osteocyte apoptosis and bone remodeling during OTM [49]. However, Moin et al. reported that osteocyte death (TUNEL-positive cells) peaked at 24 h, preceding the initial significant osteoclast formation observed at 72 h in a mouse OTM model, whereas caspase-3 activity increased only slightly suggesting that mechanisms other than the caspase-3 pathway may be involved in osteocyte death during OTM [50]. A recent study investigated OTM using the bisphosphonate analog IG9402, which specifically inhibits osteocyte apoptosis without affecting osteoclasts. The results showed that the suppression of osteocyte apoptosis altered the morphology of osteoclasts and macrophages but did not significantly influence the amount of tooth movement after 10 days in a mouse OTM model. These findings suggest that osteocyte apoptosis plays a key role in local inflammation but is not a primary determinant of tooth movement [51]. Overall, these results clearly demonstrate that osteocytes undergo cell death during OTM. However, whether osteocyte apoptosis directly contributes to bone remodeling and tooth movement remains controversial. Apoptosis is unlikely to be the primary stimulus for osteoclast formation because it induces minimal inflammatory signaling owing to the formation of membrane-enclosed apoptotic bodies [52]. Therefore, osteoclast activation and subsequent alveolar bone resorption on the compression side during OTM may be primarily mediated by necrotic, rather than apoptotic, osteocytes.

Apoptosis due to orthodontic force extends not only to osteocytes but also to osteoblasts and osteoclasts. An in vitro study on human osteoblast-like cells (MG-63 cells) demonstrated that a continuous compressive force induced apoptosis in a time- and force-dependent manner. Mechanistically, this apoptotic process is mediated by the activation of the caspase-8 signaling cascade, which subsequently promotes caspase-3 activity [53]. A study using primary osteoblasts from neonatal rat calvaria revealed that a light tensile force (6% elongation) had a protective effect, attenuating serum deprivation-induced apoptosis by downregulating caspase-8 and elevating the anti-apoptotic gene Bcl-2. Conversely, a heavy tensile force (12% elongation) promoted apoptosis with elevated caspase-3 activity [54]. Apoptosis is reported to occur in the adherent cells of bone marrow cell culture when they are cultured with both TNF-α and IL-12. The mechanism of the apoptosis is that TNF-α induces Fas expression on osteoclast precursors and IL-12 induces FasL on the non-adherent cells in bone marrow cells cultured with macrophage-colony stimulating factor (M-CSF) [55]. Yoshimatsu et al. also reported that IL-12 inhibited TNF-α-induced osteoclast formation by activating apoptosis in vivo [56]. Furthermore, local injection of IL-12 in a mouse model of OTM significantly reduced both tooth movement and root resorption, with several TUNEL-positive cells observed on the compression side. Because TNF-α was expressed in the compression side during OTM [57], the results suggested that apoptosis was activated to decrease excessive bone resorption and protect the root surface during tooth movement by Fas-FasL interaction [58]. These findings suggest that TNF-α is one of the important cytokines involved in apoptosis during OTM. Ovariectomized mice also show elevated TNF-α expression [59,60] and enhanced OTM [61]. Furthermore, MOPs, a surgical method involving perforation of the alveolar bone for accelerated tooth movement, also enhances TNF-α expression [62] as well as OTM [63]. These conditions promote tooth movement by enhancing TNF-α expression during OTM but may also promote apoptosis via TNF-α expression. Lima et al. demonstrated that mechanical loading upregulated the expression of IL-33 and its receptor ST2 in a mouse OTM model. Importantly, ST2-deficient (ST2^−/−^) mice showed enhanced bone resorption and an increased number of osteoclasts during OTM. The protective function of the IL33/ST2 axis was further supported by in vitro experiments, where IL-33 treatment suppressed osteoclast differentiation and activity by upregulating pro-apoptotic molecules, such as Bcl-2-associated X protein (BAX) and FAS, thereby promoting osteoclast apoptosis [64]. Expanding the investigation into external risk factors, cigarette smoke exposure was found to directly affect the life cycle of osteoclasts during OTM. Further, cigarette smoke significantly enhances mechanical force-induced bone resorption and increases osteoclast numbers in a mouse model of OTM. Mechanistically, in vitro experiments have confirmed that cigarette smoke inhibits osteoclast apoptosis via the mitochondrial reactive oxygen species (mtROS)/cytochrome C/caspase-3 signaling pathway, leading to an increased number of active osteoclasts and accelerated alveolar bone resorption [65]. Adaptive bone remodeling during OTM also requires careful regulation of apoptosis in osteoblasts and osteoclasts. Although fewer studies have focused on these cells than on osteocytes, the collective evidence suggests that the precise control of osteoblast and osteoclast apoptosis is directly involved in determining the rate and quality of bone turnover. Therefore, controlling osteoblast and osteoclast apoptosis is a potential strategy for better managing OTM and protecting the periodontal tissue.

### 2.2. Autophagy

Autophagy, derived from the Greek for “self-eating,” is a cellular process in which intercellular components such as proteins and organelles are transported to lysosomes or vacuoles for degradation. There are two forms of autophagy, macroautophagy and micro-autophagy [66]. The most studied form, macroautophagy, is initiated by a cup-shaped membrane structure called the phagophore, which bends and encloses intercellular components to form a double-membraned structure called the autophagosome. Autophagosomes subsequently fuse with lysosomes to form autolysosomes, leading to the degradation of their contents. In micro-autophagy, the membranes of endosomes or lysosomes invaginate inward to directly capture a part of the cytoplasm. Phagophore formation requires the unc-51-like kinase 1 (ULK1) complex, which triggers isolation membrane formation. This complex recruits the Class III PI3 Kinase complex (containing Beclin-1) for membrane nucleation. Autophagy-related gene 5 (ATG5) and microtubule-associated protein light chain 3 (LC3) are crucial for membrane elongation and closure, and result in mature autophagosomes [17,67]. Generally, autophagy plays an important protective role in cellular homeostasis maintenance. However, under certain conditions, such as autophagic processes or excessive activation, autophagy can result in cell death, referred to as “autophagy-dependent cell death” [68]. Therefore, the following section comprehensively reviews autophagy during OTM.

#### 2.2.1. Autophagy in PDL Cells

Most reports on autophagy during OTM have focused on the PDL cells. Chen et al. demonstrated that a compressive force activates autophagy in PDL cells during OTM. Using a mouse OTM model for in vivo and human PDL cells for in vitro experiments, they showed that autophagy negatively regulates osteoclast formation through the RANKL and osteoprotegerin (OPG) signaling pathway [69]. Furthermore, the same research group used a mouse OTM model and showed that autophagy of the PDL can slow tooth movement by suppressing inflammation. Inhibiting autophagy using 3-methyladenine (3-MA) accelerated tooth movement and increased inflammatory markers such as IL-1, IL-6, and TNF-α [70]. Conversely, a rat OTM model showed that both autophagy and the inflammatory cytokine, TNF-α were increased in the PDL following mechanical force application. These findings suggest a significant correlation between autophagy and TNF-α expression [71]. Using a transgenic mouse OTM model with green fluorescent protein (GFP)-tagged LC3, Li et al. found that autophagic activity increased in the PDL and was closely associated with inflammatory cytokine expression and osteoclast recruitment [72]. Jiang et al. also revealed a mechanism linking PDL stem cell autophagy to an inflammatory response. Mechanical force increased the expression LC3, the number of M1 macrophages and osteoclasts on the compression side of the PDL in a rat OTM model. By inducing macrophage polarization to the M1 phenotype, force-induced autophagy contributes to the inflammatory bone remodeling necessary for tooth movement [73]. Furthermore, aging has been reported to decrease autophagic activity and tooth movement. Adult rats (8-month-old) showed reduced osteogenic responses and delayed osteoclast formation compared with those in adolescent rats (6-week-old). Autophagy activation by rapamycin enhances tooth movement, increases early osteoclast formation, and partially restores osteogenic and aging-related factors to the adolescent levels [74].

Memmert et al. investigated the effect of static tensile strain on human PDL fibroblasts and found that autophagy induction was a force-dependent response. High tensile strain significantly induced autophagic flux and gene expression, whereas low tensile strain showed cell-protective properties [75]. Building on their previous work, they demonstrated that biomechanical loading conditions significantly increased the key autophagy marker, sequestosome 1, in human PDL fibroblasts and rat PDL tissues. These findings provide molecular insights into how tensile forces regulate autophagy-related adaptive stress responses in PDL cells during OTM [76]. Moreover, they found that mechanical pressure induced autophagy and significantly increased IL-6 expression in human PDL fibroblasts, whereas inhibition of autophagy using 3-MA further enhanced the IL-6 increase in human PDL fibroblasts and reduced OPG expression in osteoblasts. These results suggest that suppression of autophagy accelerates tooth movement by enhancing inflammatory signaling [77]. A previous study clarified the role of autophagy on the tension side, demonstrating its importance for the osteogenic differentiation of human PDL stem cells. Autophagy markers showed expression trends similar to those of osteogenesis-related factors in human PDL stem cells under mechanical stretching, confirming that autophagy plays a key role in bone formation on the tension side during OTM [78]. Tensional force induces the release of cellular communication network factor 1 (CCN1) from human periodontal ligament (PDL) stem cells, which subsequently promotes osteogenic differentiation by upregulating osteogenic markers. This osteogenic response is mediated by interlinks between the MAPK/ERK signaling pathway and autophagy [79]. To further clarify the mechanism on the tension side, Shao et al. demonstrated that cyclic tensile stress significantly enhances the osteogenic differentiation of PDL stem cells, leading to increased alkaline phosphatase (ALP) and calcium nodule formation. This enhanced bone formation is linked to the activation of mitochondrial autophagy (mitophagy), suggesting that mitophagy provides critical support for bone formation during OTM [80].

Several studies have begun to delineate the detailed mechanisms controlling mechanical force-induced autophagy in PDL cells. An investigation of long non-coding RNAs (lncRNAs) in PDL stem cells has revealed that compressive force upregulates the lncRNA Fer-1-like family member 4 (FER1L4), which subsequently induces autophagy. These findings reveal a novel lncRNA mechanism that regulates PDL stem cell autophagy during OTM [81]. A study using three-dimensional (3D)-cultured human PDL cells showed that compressive stress rapidly and transiently induces autophagy. This induction is regulated by the integrin-linked kinase (ILK) and phosphatidylinositol 3 kinase (PI3K) pathway [82]. Salim et al. identified the expression and functional relevance of chaperone-assisted selective autophagy (CASA) machinery genes in human PDL cells and rat OTM models. Their findings suggest that CASA is a central and crucial mechanism for cellular stress protection and adequate adaptation to the mechanical forces exerted during OTM [83]. Further research explored the roles of Rho kinases (ROCK) and ROS in pressure-induced autophagy in human PDL cells. Inhibition of ROCK (Y-27632) significantly reduced autophagy at all the tested pressures. The ROS scavenger N-acetylcysteine also reduced autophagy at low pressures, suggesting that both ROCK and ROS critically influenced the pressure-induced autophagic response [84]. Additionally, mechanical loading significantly upregulated damage-regulated autophagy modulator 1 (DRAM1) expression in PDL cells and a rat OTM model. This regulation of DRAM1 is dependent on the duration and magnitude of biomechanical loading and autophagy-associated pathways, suggesting a key function in force mechanotransduction [85].

The autophagy and apoptosis signaling pathways are considerably interconnected through various crosstalk mechanisms [86]. A dynamic interaction between autophagy and apoptosis has also been suggested in a rat OTM model. Autophagy markers (Beclin-1 and LC3-II) peak 1 h after OTM, with an increase in apoptosis markers (BAX and caspase-3) at 1 week after OTM [87]. Expanding on this concept, Li et al. evaluated the effects of apocynin, a plant-derived bioactive compound, on compressive force-induced apoptosis and autophagy in PDL stem cells. Apocynin treatment effectively inhibited this increase in both apoptosis and autophagy. These results suggest that apocynin can improve orthodontic outcomes by attenuating excessive apoptosis in PDL cells via regulation of the autophagy pathway [88].

Recently, several approaches have been explored to regulate autophagy during OTM in order to control tooth movement and its potential side effects. Sanhuang decoction treatment in a rat model of OTM was reported to reduce PDL damage and inhibit PDL fibroblast autophagy by reducing oxidative stress and activating the PI3K-Akt-mTOR pathway [89]. The gaseous mediator hydrogen sulfide (H_2_S) has been shown to increase mouse PDL cell autophagy and promote bone remodeling, particularly at a concentration of 0.5 mM in the mouse OTM model. This resorption-promoting and autophagy-enhancing effect is mediated by the Hippo-YAP signaling pathway, suggesting that H_2_S and its downstream targets constitute a critical axis for regulating OTM kinetics [90]. Furthermore, stem cell therapy utilizing autophagy modulation shows promise for alveolar bone regeneration. Zong et al. developed a biomimetic transplantation method using human PDL stem cells pretreated with gold nanocomplexes embedded in a collagen hydrogel. This transplant significantly prevented alveolar bone resorption in a rat OTM model by activating osteogenesis associated with the autophagy pathway [91]. Building on this strategy, the same group later engineered a biomimetic thermoresponsive hydrogel encapsulating PDL stem cells that slowly released the autophagy inducer rapamycin. This advanced system successfully prevented orthodontically induced inflammatory root resorption in a rat OTM model by enhancing PDL stem cell survival and function under stressful conditions through autophagy modulation [92]. Taken together, these results highlight autophagy as a pivotal, force-sensitive cellular mechanism in PDL cells that mediates adaptive responses to mechanical stress during OTM. The dual role of autophagy, which supports inflammatory bone resorption on the compression side and promotes osteogenesis on the tension side, makes it a critical target for regulating OTM efficiency and minimizing potential side effects.

#### 2.2.2. Autophagy in Cementoblasts

Cementoblast migration is vital for repairing root resorption during OTM. Compressive force has been shown to inhibit cementoblast migration by suppressing autophagic activity. In murine cementoblasts (OCCM-30 cells), compression reduces the expression and secretion of matrix metalloproteinase (MMP) 2, MMP 9, and MMP 13 expression and secretion without affecting apoptosis or proliferation. Activation of autophagy using rapamycin partially restores cementoblast migration [93]. Further research by the same group revealed that compressive force inhibited the mineralization capability of cementoblasts, a process mediated by autophagy suppression. This suppression involves mechanical force-induced upregulation of LincRNA-p21, which inhibits the autophagic process by binding to the transcription factor forkhead box O3 (FoxO3). Knockdown of LincRNA-p21 rescued the autophagic process in a mouse OTM model and enhanced cementogenesis by reducing root resorption [94]. Additionally, inhibition of cementoblast mineralization under compression was shown to be mediated by the autophagy-dependent regulation of periostin/β-catenin axis [95]. Conversely, when cementoblasts were subjected to mechanical tension, their autophagic activity was enhanced, leading to promotion of mineralization in a force-dependent manner. Similar to the compression side, tension-induced mineralization promotion was also mediated by periostin, linking autophagy with enhanced cementogenesis on the tension side of the tooth root [96]. Further focusing on the selective autophagy pathway, a heavy compression force was demonstrated to inhibit cementoblast mineralization and trigger root resorption by suppressing mitophagy through the downregulation of PINK and PARKIN. Mechanistically, activation of the sphingosine-1-phosphate receptor 1 (S1PR1)/mitophagy axis restored mineralization and reduced root resorption in vivo, establishing this pathway as a critical regulator of cementoblast function [97]. Overall, these findings suggest that the regulation of cementoblast autophagy is a potential therapeutic strategy for reducing root resorption during OTM.

#### 2.2.3. Autophagy in Bone-Related Cells

Recent studies have revealed that autophagy plays a pivotal role in the osteocyte-mediated responses to mechanical stress during OTM. Under compressive force, osteocyte autophagy is activated via transcription factor E3 (TFE3)-associated signaling, leading to increased RANKL secretion. The number of LC3B-potive osteocytes is markedly elevated on the compression side in a murine OTM model, and both mechanical loading and pharmacological activation of autophagy enhance RANKL expression and osteoclast formation [98]. Conversely, under tensile force, osteocyte autophagy is upregulated through AMPK-dependent pathways, thereby promoting osteogenic signaling. Enhanced autophagic activity increases the secretion of fibroblast growth factor 23 (FGF23), and conditioned media from tensioned osteocytes stimulate osteoblast proliferation and differentiation [99]. Collectively, these findings indicate that osteocyte autophagy exerts dual regulatory effects on bone remodeling during OTM, facilitating osteoclast formation under compression and promoting osteogenesis under tension, thereby orchestrating a balanced bone turnover in response to mechanical loading.

Macrophages and osteoclast lineage cells also show autophagy-dependent responses to mechanical loading during OTM. Orthodontic force induces autophagy in a force-dependent and cell type-specific manner, particularly within macrophages and osteoclasts in a murine OTM model, correlating with the altered expression of bone turnover and inflammatory markers [100]. Under compressive stress, activation of the NLRP3 inflammasome via the cyclic GMP-AMP synthase (cGAS)/purinergic 2X7 receptor (P2X7R) axis promotes osteoclast formation, whereas inhibition of autophagy enhances this response, suggesting that autophagy suppresses inflammasome-driven bone resorption during OTM [101]. Pharmacologically, strontium ranelate suppresses tooth movement and root resorption by downregulating the autophagy of osteoclast precursors through NF-κB-dependent signaling, an effect partially reversed by rapamycin-induced autophagy activation [102]. Together, these findings highlight that autophagy in macrophages and osteoclast lineage cells is a critical modulator of alveolar bone resorption during OTM, acting as a balance between inflammatory activation, osteoclast differentiation, and the rate of tooth movement.

In addition to osteocytes and macrophages, bone marrow-derived mesenchymal stem cells (BMSCs) also rely on autophagic regulation during OTM. In a beagle dog OTM model, Zhu et al. reported that orthodontic pressure initially activated autophagy in BMSCs but gradually suppressed it with prolonged loading, concomitant with increased apoptosis and reduced osteogenic differentiation, suggesting an inverse relationship between autophagy and apoptosis in maintaining osteogenesis [103]. Further, Huang et al. demonstrated that under osteoporotic conditions in a murine OTM model, lithium chloride restored autophagic activity, reduced apoptosis, and promoted bone formation, thereby protecting against excessive tooth movement and bone loss [104].

Collectively, these findings indicate that autophagy in bone-related cells orchestrates both osteoclastic and osteogenic dynamics and acts as a central regulatory mechanism for maintaining alveolar bone homeostasis during OTM.

### 2.3. Pyroptosis

Pyroptosis is a form of RCD derived from the Greek term “pyro,” meaning fire or heat. This type of cell death was initially identified as caspase-1-dependent cell death in macrophages infected with *Salmonella typhimurium* and was formally designated “pyroptosis” by Brad Cookson and Molly Brennan in 2001 [105]. During bacterial infection, macrophages undergo pyroptosis in response to pathogen-associated molecular patterns (PAMPs), which are recognized by Toll-like receptors (TLRs) among pattern-recognition receptors (PRRs) [106]. Pyroptosis can also be induced by various other stimuli, including damage-associated molecular patterns (DAMPs) [107]. These signals promote the assembly of the inflammasome, a multiprotein complex composed of PRRs and caspase-1 [108]. Pyroptosis is mediated by gasdermin D (GSDMD), which forms membrane pores and concomitantly enables the release of pro-inflammatory cytokines such as IL-1β and IL-18 [3,109,110].

A recent comprehensive review established that inflammasome activation and downstream pyroptosis play key roles in driving alveolar bone loss in etiologically diverse diseases [111]. Although not associated with pyroptosis, Yan et al. initially reported changes in caspase-1 expression in rat PDL tissues during OTM. They found that orthodontic force significantly upregulated caspase-1 expression in rat PDL tissues, peaking at approximately day 3 [112]. Maruyama et al. demonstrated that cyclic tensile force, a type of mechanical stress, inhibited the pyroptotic pathway in murine macrophages in vitro. Specifically, they found that cyclic force suppressed NLRP3 inflammasome activation, resulting in IL-1β secretion by attenuating caspase-1 activation via the AMPK pathway [113]. Further connecting systemic factors to this pathway, Chen et al. found that diabetes mellitus exacerbated alveolar bone loss and inflammation during OTM in rats. They demonstrated that high glucose and mechanical force activated the NLRP3 inflammasome pathway in PDL fibroblasts. This activation was confirmed by the increased expression of NLRP3, caspase-1, GSDMD, and IL-1β, suggesting the involvement of NLRP3 inflammasome in diabetes-induced periodontal pathology [114]. Subsequently, mechanical force was directly confirmed to induce caspase-1-dependent pyroptosis in PDL progenitor cells, promoting alveolar bone remodeling and tooth movement. This study showed that modulating pyroptosis directly suppressed or promoted OTM. Mechanistically, this force-induced pyroptosis influences osteoclast formation and is mediated by transient receptor potential subfamily V member 4 signaling [115]. Finally, mechanical force induced caspase-1-dependent pyroptosis in macrophages was demonstrated to trigger sterile inflammation in a rat OTM model. Mechanistically, force disrupts cellular energy metabolism by creating an imbalance between lactate dehydrogenase A (LDHA) and pyruvate dehydrogenase (PDH), leading to mitochondrial dysfunction. Notably, inhibition of pyruvate dehydrogenase kinase 1 (PDK1) restores metabolic balance and alleviates pyroptosis in these force-stimulated macrophages, thereby revealing a novel therapeutic target [116].

Pyroptosis is also suggested to be involved in root resorption during OTM. The NLRP3 inflammasome and M1 macrophages are known to be expressed around the root resorption areas induced by excessive force in the rat OTM model. Force-loading human PDL cells promote M1 macrophage polarization, NLRP3 activation, and subsequent IL-1β production in vitro [117]. Further mechanisms linking PDL cells to root resorption have also been elucidated. RNA sequencing revealed that excessive force transduces inflammatory signals via the TLR4/NFκB/NLRP3 pathway, promoting root resorption via NLRP3-mediated PDL cell pyroptosis. NLRP3 inhibition reduces root resorption, odontoclast formation, and M1 polarization, confirming that pyroptotic products from PDL cells directly and indirectly accelerate root resorption [118]. A potential therapeutic strategy was explored, demonstrating that exosomes derived from dental follicle stem cells (DFSC-Exos) reduced root resorption by inhibiting PDL cell pyroptosis. DFSC-Exos inhibited NLRP3-mediated pyroptosis, subsequent M1 polarization, and osteoclast formation. DFSC-Exos transfer miR-140-3p to PDL cells, which blocks the DNA methyltransferase 1 (DNMT1)/suppressor of cytokine signaling (SOCS1)/NFκB axis to downregulate the pyroptosis pathway, thereby reducing root resorption [119]. Recently, the epigenetic regulation of pyroptosis during root resorption has been elucidated. HDAC9-mediated histone deacetylation induces mitochondrial dysfunction and pyroptosis in human PDL fibroblasts by reducing H3K9ac enrichment on mitochondrial pathway genes. This HDAC9-mediated pyroptosis promotes osteoclast differentiation and accelerates root resorption, thus establishing a novel link between epigenetic modifications and inflammatory cell death [120].

Collectively, pyroptosis is not merely a consequence of cellular stress, but also a central, mechanistically regulated inflammatory driver of OTM-induced pathology, suggesting promising approaches for accelerating tooth movement and mitigating adverse outcomes like root resorption.

### 2.4. Ferroptosis

Ferroptosis is an iron-dependent form of RCD first described in 2012 by Brent Stockwell, and its name is derived from the Latin word “ferrum,” meaning iron [121]. This type of cell death is characterized by the depletion of intracellular glutathione and reduced activity of glutathione peroxidase 4 (GPX4), which together result in the accumulation of unmetabolized lipid peroxides and increased production of ROS, ultimately disrupting the plasma membrane [3,122]. Ferroptosis has been implicated in various physiological conditions including ischemic injury, cancer, and neurodegenerative diseases [123,124]. Recent comprehensive reviews have established ferroptosis as a novel and intriguing concept relevant to the pathophysiology of several oral diseases, including periodontitis, pulpitis, and oral cancer [125].

Ferroptosis is implicated in the alveolar bone loss observed in OTM. Mechanical stress has been shown to induce GPX4-dependent ferroptosis in osteoblasts on the compression side during OTM. Ferroptosis also promotes alveolar bone loss by activating the YAP-transcriptional enhanced associate domain (TEAD) signaling pathway, leading to decreased bone formation. Crucially, the ferroptosis inhibitor Ferrostatin-1 was successfully shown to inhibit bone loss in vivo, highlighting the GPX4 and YAP pathways as potential therapeutic targets for tooth movement [126]. The role of ferroptosis extends to accelerated tooth movement models, such as corticotomy. Single-cell RNA sequencing of the alveolar bone after corticotomy revealed a significant increase in iron metabolism-related genes in macrophages. Pseudotime and SCENIC analyses identified a new macrophage developmental state characterized by the transcription factor Atf3, which was closely associated with osteoclasts and increased cell communication. These findings suggest that Aft3-positive macrophages, which are potentially linked to ferroptosis via altered iron metabolism, play a key role in corticotomy-accelerated tooth movement [127]. Despite these findings, the specific role of ferroptosis in orthodontically induced inflammatory root resorption remains unclear. A recent review hypothesizes that ferroptosis likely contributes to root resorption pathogenesis during OTM because of its close association with inflammation and bone homeostasis. However, direct evidence clarifying the modulatory mechanisms of ferroptosis in root resorption during OTM is still required to develop targeted therapeutic strategies [128]. Taken together, ferroptosis is an active, mechanistically RCD critical for bone remodeling during tooth movement.

### 2.5. Necroptosis

Necroptosis is another form of RCD that exhibits morphological features similar to those of classical necrosis. Research on this pathway began with the observation of TNF-α-induced necrotic cell death, leading to its formal identification in 2005 using Necrostatin-1, an inhibitor of receptor-interacting protein (RIP) 1 [129]. Necroptosis typically involves the sequential phosphorylation of RIP1, RIP3, and mixed lineage kinase domain-like protein (MLKL). This process results in rupture of the plasma membrane and subsequent release of DAMPs [3,52]. The released DAMPs drive sterile inflammation and are implicated in various pathological conditions, including bone diseases, such as rheumatoid arthritis and osteoarthritis [130,131].

The role of necroptosis in regulating the bone remodeling process specific to OTM has recently been elucidated. Our research group previously investigated the effects of osteocyte necroptosis on osteoclast formation during OTM in mice. We found that orthodontic force significantly increased osteocyte death, particularly on day 6. The presence of necroptosis was confirmed by detecting the markers p-RIP3 and p-MLKL in osteocytes on the compression side. This finding was notably absent in TNF receptor-deficient mice. Overall, osteocyte necroptosis enhances osteoclast formation in vitro, suggesting that necroptotic osteocytes drive alveolar bone resorption by releasing inflammatory factors such as DAMPs during OTM [132]. Presence of the inflammatory cytokine TNF-α during OTM is well established [12,57,133]. As TNF-α is generally expressed in the PDL environment, it is highly likely that necroptosis also occurs in cells other than osteocytes. However, further investigation is required to fully elucidate the role of necroptosis in different cells and its regulation of alveolar bone remodeling. Clarifying these mechanisms will facilitate a deeper understanding of inflammation in OTM and may provide novel therapeutic strategies for controlling tooth movement.

## 3. Summary and Limitation

**Figure 2:**
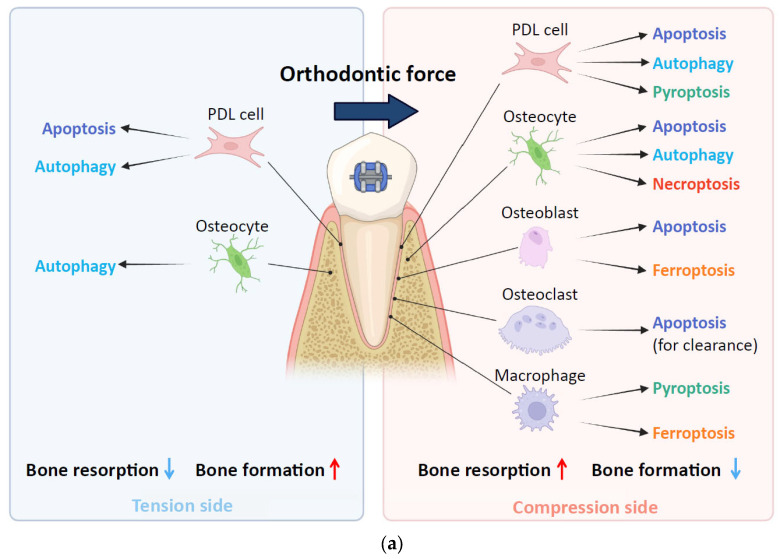

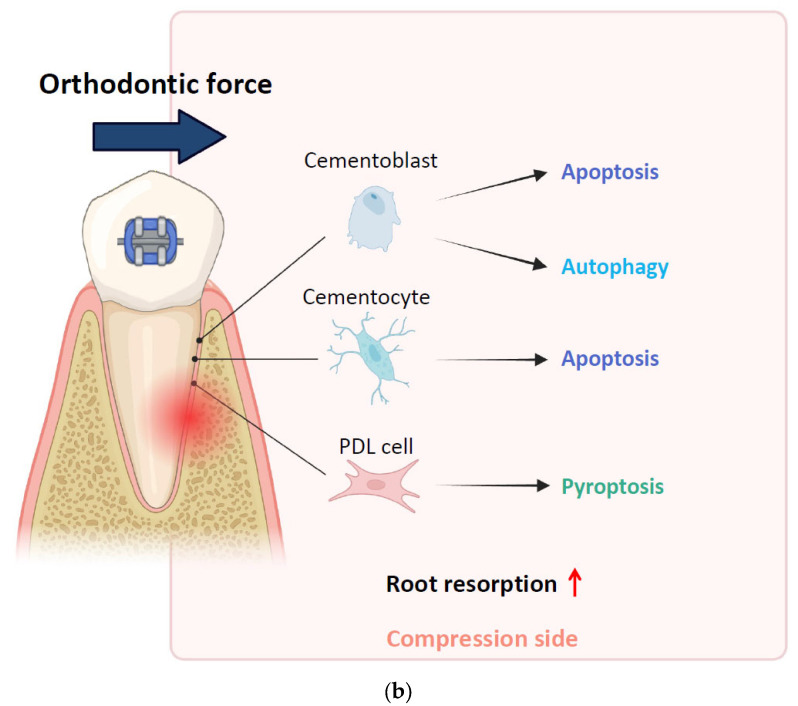
Diverse roles of regulated cell death (RCD) in alveolar bone remodeling and root resorption during orthodontic tooth movement (OTM). (**a**) Cellular mechanisms of alveolar bone remodeling. On the compression side (red panel), various forms of RCD drive alveolar bone resorption and suppress bone formation. On the tension side (blue panel), apoptosis and autophagy primarily promote osteogenic differentiation and bone formation. (**b**) Mechanisms of RCD induced root resorption. On the compression side, mechanical stress triggers RCD pathways—including apoptosis, autophagy, and pyroptosis—which collectively contribute to the environment promoting root resorption. Created in BioRender. F.O. (2025). https://BioRender.com (accessed on 26 November 2025).

Tooth movement induced by orthodontic force is achieved through strictly well-regulated alveolar bone remodeling with diverse RCD, moving far beyond the classical concepts of simple apoptosis and necrosis (Figure 2). The cellular response to mechanical forces is highly cell type- and location-specific. On the compression side, the RCD primarily promotes alveolar bone resorption. Apoptosis of PDL cells is an early and crucial event preceding bone resorption. Pyroptosis in PDL cells acts as a potent inflammatory driver of alveolar bone resorption and potential root resorption. Ferroptosis in osteoblasts contributes to bone remodeling by suppressing bone formation. Furthermore, necroptosis in osteocytes promotes osteoclast formation and alveolar bone resorption largely through the release of DAMPs. The role of autophagy on the compression side is complex, acting as an adaptive stress response that can both regulate inflammatory signaling in PDL cells and promote osteoclast formation via osteocyte signaling, whereas autophagy suppression in cementoblasts leads to impaired repair and increased root resorption. Conversely, RCD often supports tissue regeneration on the tension side. Autophagy in both PDL stem cells and osteocytes primarily promotes osteogenic differentiation and bone formation.

A critical evaluation of the current literature reveals significant controversies, particularly regarding the role of autophagy in OTM. While some studies suggest that autophagy acts as an anti-inflammatory “brake” that slows tooth movement [70,77], others identify it as a pro-inflammatory “accelerator” that promotes macrophage polarization and osteoclastogenesis [72,73]. These conflicting findings likely stem from differences in force magnitude (light vs. heavy) and the specific cell types observed. Furthermore, the transition from apoptosis to necroptosis or necrosis in osteocytes appears to be a key determinant of the inflammatory intensity on the compression side. This comparative analysis suggests that the biological outcome of OTM is not merely a product of “cell death” in general but is finely tuned by the specific RCD pathway activated under different mechanical conditions. A fundamental question remains whether cell death is the primary initiator of bone remodeling or a secondary consequence of mechanical stress. The evidence reviewed here suggests a dual role. Early-phase RCD, such as PDL cell apoptosis [22] and osteocyte necroptosis [132], appears to function as a primary initiator by releasing signaling molecules that actively recruit and activate bone-remodeling cells. In contrast, late-stage cell death, often seen in cementoblasts or during hyalinized tissue formation under excessive force, may represent a secondary consequence of hypoxia and physical trauma. Distinguishing between these primary regulatory deaths and secondary pathological deaths is crucial for developing targeted strategies to optimize tooth movement while minimizing root resorption. A comparative summary of these specific cell death pathways, their roles, and functional impacts is provided in Table 1.

However, this review has several limitations that should be considered. Many studies investigating cell death mechanisms during OTM have primarily used animal models, such as rats, mice, and dogs. The biological responses and cellular pathways in these animal species may differ from those in humans. Therefore, the direct translation of findings from these animal models to human clinical situations may not always be straightforward. Furthermore, the methods used to detect specific types of cell death present challenges. For instance, TUNEL staining, which is commonly employed to identify apoptosis, is known to potentially yield positive results for other forms of cell death as well. This makes it difficult to precisely determine the specific predominantly active cell death mechanism, leading to the need for more precise and definitive detection techniques in future research.

## 4. Conclusions and Future Directions

To synthesize our findings, each RCD pathway exerts a distinct functional impact: apoptosis serves as a non-inflammatory initiator of tissue turnover; autophagy acts as a metabolic rheostat balancing bone formation and resorption; pyroptosis and necroptosis function as potent pro-inflammatory drivers of osteoclastogenesis; and ferroptosis contributes to the suppression of bone formation under excessive stress. The integration of these pathways suggests that OTM is not merely a result of mechanical injury, but a highly programmed biological response. The coordinated spatial and temporal regulation of the five major RCD pathways, apoptosis, autophagy, pyroptosis, ferroptosis, and necroptosis, across the key cellular populations involved in OTM (PDL cells, cementoblasts, cementocytes, and bone-related cells), plays a central role in determining both the biological efficiency of tooth movement and the risk of pathological sequelae, such as root resorption. Emerging evidence indicates that targeting specific molecular regulators of these cell death pathways may offer a promising approach for developing safer, more precise, and predictable strategies for orthodontic treatment.

For future research, we propose three key methodological directions to enhance the practical value of these findings. First, advanced spatio-temporal mapping, such as single-cell RNA sequencing and spatial transcriptomics, is required to pinpoint the exact transition from physiological to pathological cell death. Second, the development of localized drug-delivery systems, such as biomimetic hydrogels, should be prioritized to modulate specific RCD pathways clinically. Finally, future studies must transition from non-specific assays like TUNEL to more definitive molecular markers, such as GSDMD or phosphorylated MLKL, to ensure precise identification of RCD types. These research objectives will provide clear guidelines for translating molecular insights into improved orthodontic treatment.

## Figures and Tables

**Table 1:** Comparative analysis of major RCD pathways during OTM.

Cell Type	RCD	Side	Role (Primary vs. Secondary)	Functional Impact on OTM	Key Ref.
PDL cell	Apoptosis	Comp.	Primary	Initiates bone resorption	[22]
	Autophagy	Comp.	Controversial	Dual role	[70,73]
	Pyroptosis	Comp.	Primary	Promotes bone and root resorption	[115,118]
Osteocyte	Apoptosis	Comp.	Secondary	Local inflammation: role of OTM debated	[51]
	Autophagy	Comp.	Primary	Increase RANKL secretion	[98]
	Necroptosis	Comp.	Secondary	Releases DAMPs; triggers resorption	[132]
Osteoblast	Ferroptosis	Comp.	Primary	Suppress bone formation	[126]
Cementoblast	Autophagy	Comp.	Secondary	Impairs repair; promotes root resorption	[93,94]
PDL cell	Autophagy	Tens.	Primary	Promotes osteogenic differentiation	[78,80]

## Data Availability

No new data were created or analyzed in this study. Data sharing is not applicable to this article.

## Abbreviations

The following abbreviations are used in this manuscript:

PCD	Programmed cell death
ACD	Accidental cell death
RCD	Regulated cell death
NCCD	Nomenclature Committee on Cell Death
MPT	Mitochondrial permeability transition
OTM	Orthodontic tooth movement
Bcl-2	B-cell lymphoma 2
TNF	Tumor necrosis factor
FAS	FS-7-associated surface antigen
TUNEL	Terminal deoxynucleotidyl transferase-mediated dUTP nick end labeling
PDL	Periodontal ligament
PCNA	Proliferating cell nuclear antigen
ROS	Reactive oxygen species
MOPs	Micro-osteoperforations
SOD2	Superoxide dismutase 2
BIRC3	Baculoviral IAP repeat-containing protein 3
Lepr+	Leptin receptor-positive cells
IL	Interleukin
PDGF	Platelet-derived growth factor
RANKL	Receptor activator of nuclear factor-kappa B
HIF-1α	Hypoxia inducible factor 1α
CTGF	Connective tissue growth factor
M-CSF	Macrophage-colony stimulating factor
BAX	Bcl-2-associated X protein
mtROS	Mitochondrial reactive oxygen species
ULK1	Unc-51-like kinase 1
ATG5	Autophagy-related gene 5
LC3	Microtubule-associated protein light chain 3
OPG	Osteoprotegerin
3-MA	3-Methyladenine
GFP	Green fluorescent protein
CCN1	Cellular communication network factor 1
ALP	Alkaline phosphatase
lncRNAs	Long non-coding RNAs
FER1L4	Fer-1-like family member 4
ILK	Integrin-linked kinase
PI3K	Phosphatidylinositol 3 kinase
CASA	Chaperone-assisted selective autophagy
ROCK	Rho kinases
DRAM1	Damage-regulated autophagy modulator 1
H_2_S	Hydrogen sulfide
MMP	Matrix metalloproteinase
FoxO3	Forkhead box O3
S1PR1	Sphingosine-1-phosphate receptor 1
TFE3	Transcription factor E3
AMPK	AMP kinase
FGF23	Fibroblast growth factor 23
NLRP3	NOD-like receptor family pyrin domain-containing 3
cGAS	Cyclic GMP-AMP synthase
P2X7R	Purinergic 2X7 receptor
BMSC	Bone marrow-derived mesenchymal stem cell
PAMPs	Pathogen-associated molecular patterns
TLRs	Toll-like receptors
PRRs	Pattern-recognition receptors
DAMPs	Damage-associated molecular patterns
GSDMD	Gasdermin D
LDHA	Lactate dehydrogenase A
PDH	Pyruvate dehydrogenase
PDK1	Pyruvate dehydrogenase kinase 1
DFSC-Exos	Exosomes derived from dental follicle stem cells
DNMT1	DNA methyltransferase 1
SOCS1	Suppressor of cytokine signaling
GPX4	Glutathione peroxidase 4
TEAD	Transcriptional enhanced associate domain
RIP	Receptor-interacting protein
MLKL	Mixed lineage kinase domain-like protein
